# Logistical constraints lead to an intermediate optimum in outbreak response vaccination

**DOI:** 10.1371/journal.pcbi.1006161

**Published:** 2018-05-23

**Authors:** Yun Tao, Katriona Shea, Matthew Ferrari

**Affiliations:** Department of Biology and The Center for Infectious Disease Dynamics, The Pennsylvania State University, University Park, Pennsylvania, United States of America; Emory University, UNITED STATES

## Abstract

Dynamic models in disease ecology have historically evaluated vaccination strategies under the assumption that they are implemented homogeneously in space and time. However, this approach fails to formally account for operational and logistical constraints inherent in the distribution of vaccination to the population at risk. Thus, feedback between the dynamic processes of vaccine distribution and transmission might be overlooked. Here, we present a spatially explicit, stochastic Susceptible-Infected-Recovered-Vaccinated model that highlights the density-dependence and spatial constraints of various diffusive strategies of vaccination during an outbreak. The model integrates an agent-based process of disease spread with a partial differential process of vaccination deployment. We characterize the vaccination response in terms of a diffusion rate that describes the distribution of vaccination to the population at risk from a central location. This generates an explicit trade-off between slow diffusion, which concentrates effort near the central location, and fast diffusion, which spreads a fixed vaccination effort thinly over a large area. We use stochastic simulation to identify the optimum vaccination diffusion rate as a function of population density, interaction scale, transmissibility, and vaccine intensity. Our results show that, conditional on a timely response, the optimal strategy for minimizing outbreak size is to distribute vaccination resource at an intermediate rate: fast enough to outpace the epidemic, but slow enough to achieve local herd immunity. If the response is delayed, however, the optimal strategy for minimizing outbreak size changes to a rapidly diffusive distribution of vaccination effort. The latter may also result in significantly larger outbreaks, thus suggesting a benefit of allocating resources to timely outbreak detection and response.

## Author summary

It has long been recognized that an epidemic of infectious disease can be prevented if a sufficient proportion of the susceptible population is vaccinated in advance. This logic also holds for vaccine-based outbreak response to stop an outbreak of a novel, or re-emerging pathogen, but with an important caveat. If vaccination is used in response to an outbreak, then it will necessarily take time to achieve the required level of vaccination coverage, during which time the outbreak may continue to spread. Thus, one must consider the logistical and operational constraints of vaccine distribution to assess the ability of outbreak response vaccination to slow or stop an advancing epidemic. We develop a simple mathematical framework for representing vaccine distribution in response to an epidemic and solve for the optimal distribution strategy under realistic constraints of total vaccination effort. Focused deployment near the outbreak epicenter concentrates resources in the area most in need, but may allow the outbreak to spread outside of the response zone. Broad deployment over the whole population may spread vaccination resources too thin, creating shortages and delays at the local scale that fail to prevent the advancing epidemic. Thus we found that, in general, the best strategy is an intermediate optimum that deploys vaccine neither too slow to prevent escape from the outbreak epicenter, nor too fast to spread resources too thin. The specific optimum rate for any given outbreak depends on the infectiousness of the pathogen, the population density, the range of contacts amongst individuals, the timeliness of the response, and the vaccine intensity. This insight only emerges from linking an epidemic model with a realistic model of outbreak response and highlights the need for further work to merge operations research with epidemic models to develop operationally relevant response strategies.

## Introduction

In applied epidemiology, models are increasingly used to inform management decisions on effective responses to a variety of outbreaking diseases, e.g. cholera [1], smallpox [2], influenza [3], Ebola [4], measles [5], and Zika fever [6]. In general, these epidemic dynamic models are designed with an emphasis on the transmission process, which includes factors such as individual encounter rates resulting from social interaction structure [7], cultural practices (e.g. funerals [8]), municipal layout (e.g. availability of hospitals [9]), transportation network [10]; seasonal aggregation [11–12], and vector behavior and abundance [13]. These types of models are commonly used to evaluate the potential impact of various interventions at the population scale, e.g. vaccination parameters in SEIR model [14], or local scale, e.g. probability of livestock on farms being culled [15]. By comparison, the dynamics of interventions have received much less attention. Here, we explicitly model vaccination response to an outbreak, and examine the interactions between disease dynamics and the impact of an intervention strategy.

Density-dependent pathogen transmission is a standard assumption in most disease models, represented by a positive correlation between local population density and the probability of individual infection. Few studies, however, consider the effect of density-dependence on outbreak control. In compartmental models of rubella (e.g. [16–17]), vaccination has been described simply as a rate that individuals are removed from the total susceptible pool. Similarly, in early individual-based, foot-and-mouth models that evaluate livestock culling strategies (e.g. [18–19]), farms or infected premises are culled at a rate independent of the total number of farms to be culled or the number of its neighboring farms designated for culling (but see [20–22] for the incorporation of logistical constraints). We propose a simple control model that features a more realistic assumption: for a given amount of vaccination effort, it takes longer to reach all susceptibles in densely populated locations.

The utility of a disease response model arguably hinges on its potential to inform management decisions for uncertain future outbreak scenarios under known logistical constraints. Realistic limitations on controls have been frequently neglected in disease transmission models in the interest of analytical simplicity, leading to assumptions of a spatially constant vaccination rate (e.g. [23]), a nearly immediate and uniform coverage of control areas (e.g. [24]), or zero wait-time before patients become vaccinated (e.g. [25]). Isolated features of control complexity can be found in a few key studies. For example, Handel et al [26] considered time-varying vaccination strategies in a well-mixed model under vaccine limitation in which the strengths of the controls were adjusted to sustain a tolerable level of effective reproduction number and thus provide protection against future outbreaks. This model considers temporal variation of the control process as a solution to disease recurrence rather than an intrinsic, logistical feature of vaccination in general. Spatially heterogeneous interventions have been investigated in heuristic models of smallpox and cholera interventions [27–28], however, vaccines are commonly assumed to be administered homogenously within the control zones (e.g. rings, discrete patches) and at rates invariant to local demands. In contrast to heuristic representations of disease response, there exist large-scale stochastic simulation models such as NAADSM (North American Animal Disease Spread Model), InterSpread Plus, and Exodis designed to replicate explicit control measures (e.g. daily farm destruction capacity) with delayed and spatially heterogeneous effects [29]. These models are better able to account for realistic logistical limitations, but the high dimensionality of these models makes it difficult to abstract their context-specific results to general control settings. Here, we explicitly model the general dynamics between transmission and vaccination intervention and account for their real time interactions. Specifically, we consider a vaccination response that is both spatially constrained and density-dependent.

As with disease transmission, vaccination may be assumed to originate from a focal location from which the response is coordinated: e.g. a central distribution point for house-to-house campaign as is used in polio vaccination or village-to-village strategies used for measles immunization days [30–32]. In the pressing case of an outbreak, the rate at which available response efforts and resources should be actively deployed into the surrounding area poses a practical scheduling challenge in operations research by public health organizations [33]. In traditional models where this deployment process occurs instantaneously, the recommended response strategies inevitably fail to account for the differential delays in vaccine delivery to different segments of the susceptible population. But in a response model with an explicit inclusion of spatial constraint, how to deploy vaccines into new, vulnerable territories may be recognized as a distribution problem for a spatially limited amount of control capital (e.g. effort, resource).

Our model is developed within an original framework that integrates an agent-based simulation of disease spread with a partial differential equation that describes the spatiotemporal distribution of vaccination effort. Prior to this study, spatial models of disease dynamics have often been developed using one of these two frameworks, which represent the Lagrangian and the Eulerian approaches, respectively. We use agent-based simulations to describe the process of transmission as a collection of random infective contacts at the individual level. Simultaneously, we characterized the vaccination response using a compartmental model based on a partial differential equation to describe the process of response as spatiotemporal variations in vaccination coverage at the population level. The combination of these approaches allows us to predict the epidemiological consequences of implementing a robust management decision in response to any realization of a novel outbreak. Our objective is to evaluate the performance of explicit vaccination strategies where the total vaccination effort is constrained on the landscape and its effectiveness varies with local population densities. In particular, we aim to identify context-specific, “optimal” vaccination strategies that minimize expected outbreak size given constrained vaccination effort.

We consider a continuum of vaccination strategies for a fixed amount of vaccination effort. On one end of this range, we have “fast and free” vaccination strategies with a high rate of radial diffusion, which cover a large area quickly, but leave few vaccinators per unit area thereby reducing local vaccination efficiencies. On the other end, we have “slow and steady” vaccination strategies with low rates of radial diffusion, which result in high local coverage but introduce an opportunity cost by delaying the implementation of vaccination efforts in areas far from the initial focus. In other words, by constraining total response effort, the former strategy ensures low intensity of vaccination broadly, while the latter results in high intensity of vaccination locally.

Here, we illustrate that the optimal strategy for vaccine distribution depends on both the population density and the promptness of the outbreak response. We find that a “one size fits all” policy does not exist; there are situations where the optimal strategy for minimizing outbreak size is to concentrate vaccination effort locally to control disease spread, and others where a rapid spatial expansion of vaccination effort is crucial for reducing overall burden. By extension, our context-specific results highlight a tension between direct and indirect protection: if a “slow and steady” strategy can contain an outbreak, then indirect protection can be realized broadly by direct protection concentrated in a small area; by contrast, a “fast and free” strategy distributes direct protection over a broad area, and the resulting indirect protection is local (i.e. neighbors) in scale.

## Methods

We define our model on a two-dimensional discretized landscape that contains *N* randomly distributed susceptible individuals. Because we consider outbreak and response dynamics on a single, fixed landscape, *N* is also a measure of population density. The first infected individual is introduced at a central location **x**_0_ at time *t* = 0, from which point disease transmission may occur at the end of every time step. At each location **x** and time *t*, the probability of infection for a susceptible individual is assumed to increase asymptotically with transmission rate *δ* and the local density of infected individuals, such that the individual becomes infected with probability

φ(x,t)=1−exp[−δI(x,t)]. (1)

The local force of infection, *I*(**x**,*t*), is defined by smoothing the distribution of infected individuals with a interaction kernel, *K*, which characterizes the likelihood of an interaction between two individuals as a function of their distance apart. We assume that the interaction kernel is a bivariate Gaussian density function with mean zero and variance-covariance matrix *α*^2^**I**, where *α*^−1^ is the rate at which interaction between individuals decays as a function of their distance apart. During each time step, an infected individual may recover with probability *ρ*.

In the numerical simulations, vaccination is carried out per time step *τ* immediately after disease transmission and before recovery. The effort available for vaccination, e.g. the quantity of operational equipment or number of healthcare personnel, at a particular space and time, *H*(**x**,*t*), is modeled as a probability surface that is initially distributed according to another bivariate Gaussian density function with mean at the disease epicenter **x**_0_, which may also represent a central distribution point or medical facility from which the response originates, and variance-covariance matrix **I**. We thus implicitly assume that vaccination effort is delivered to all areas within the control region. The probability that an individual at a particular location is immunized is assumed to increase asymptotically with vaccine intensity *ε* and the amount of available vaccination effort at that location relative to the local density of individuals who must be vaccinated. Therefore, analogous to the transmission process, a person becomes immunized at location **x** and time *t* with probability

ω(x,t)=1−exp[−εH(x,t)/S(x,t)], (2)

where local density of susceptibles, *S*(**x**,*t*), is defined by smoothing the distribution of susceptible individuals using the previous interaction kernel *K* with parameter *α* and the distribution of vaccination effort, *H*(**x**,*t*), is defined below.

We assume that the total available vaccination effort at the global scale remains constant over time but finite across space, such that ∫_Ω_*H*(**x**,*t*)*d***x** = 1, where *Ω* denotes the region over which the control may reach; here the full landscape. We maintain this conservation constraint by numerically applying zero-flux boundary condition on the landscape borders, then validate based on the integral’s deviation from unity at the end of the simulation. The process of vaccination deployment conceptually represents the collective movement activity of healthcare workers, which spreads radially into the susceptible population within the landscape boundaries. We therefore model *H*(**x**,*t*) as a two-dimensional diffusion equation:

$$\frac{\partial H}{\partial t}(x,t)=\nabla^{2}(\mu H(x,t)),$$ (3)

where diffusion rate *μ* defines the vaccination strategy. The introduction of a constant diffusion coefficient in the time derivative of *H*(**x**,*t*) assumes that the successive movement distances of healthcare are exponential and their movement direction is described by a von Mises distribution with respect to the epicenter [34]. Specifically, *μ* is proportional to the mean-squared displacement distance of vaccination effort per time step. In our present model, which aims to provide a heuristic and generalized description of disease response, we implicitly assume for *H*(**x**,*t*) that vaccination activities are informed by perfect knowledge of the disease status of all individuals at all times, such that vaccination effort is expended entirely on susceptibles, avoiding vaccination of already infected or recovered individuals. The potential consequence of information uncertainty in surveillance is beyond the scope this study.

Starting from *t* = 0, with one infected individual centrally seeded, we advance the model by time step *τ* and simulate the sequential events of outbreak and response. To obtain transient solutions of *H*(**x**,*t*), we solve Eq 3 forward in time by *τ*, using FiPy [35], a finite volume partial differential equation solver written in Python. Transmission and vaccination processes continue in succession until either the number of infected or susceptible individuals in the population drops to zero. Because the simulation terminates only when the above epidemiological condition is satisfied, the time frame is variable across replicates. The model description follows the ODD (Overview, Design concepts, and Details) protocol [36–37] (S1 Appendix).

**Fig 1:**
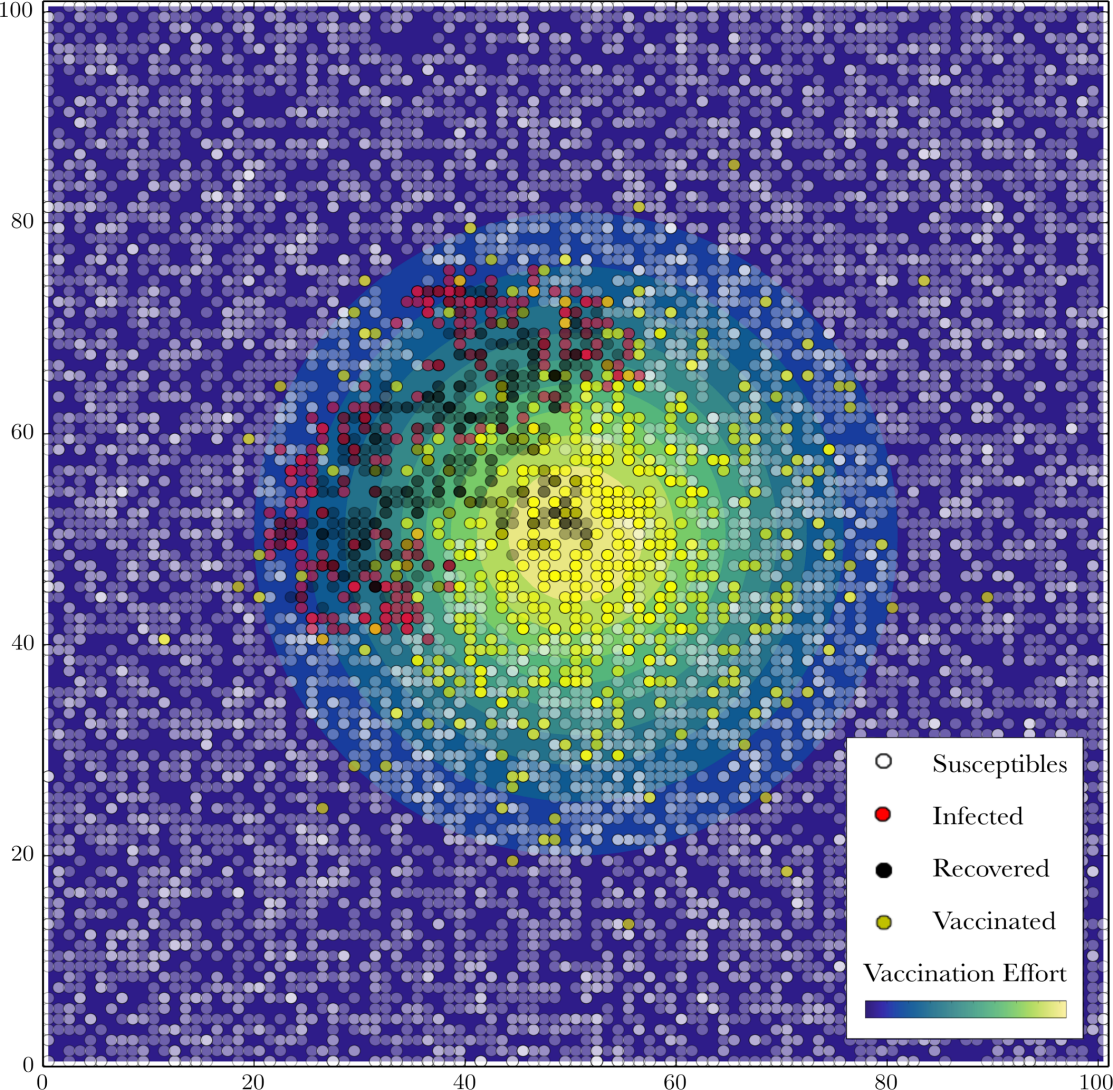
Snapshot of simulated timely vaccination response to an escaping outbreak. Image captured at time *t* = 4 in a population initialized with *N* = 10201 susceptibles, vaccination diffusion rate *μ* = 25, time step *τ =* 0.1, transmission rate *δ =* 2, recovery rate *ρ* = 0.2, vaccine intensity *ε* = 20, and interaction scale *α =* 1. See S1 Video for the full visual illustration.

We explore the effect of population density and the rate of radial expansion of a vaccination program on the number of individuals either directly protected by vaccination or indirectly protected by herd immunity. On a 101 x 101 square grid, we ran 6000 simulation replicates of an outbreak per population density, ranging from 2000 to 10201 (the latter gives an average density of one individual per grid cell) individuals on the landscape (Fig 1). Each replicate is initialized with randomly located susceptibles. Vaccination response is determined by the chosen rate of radial expansion *μ*, varying from 0.5 to 50. To clarify, based on the constraint on total vaccination effort, a slow expansion at rate *μ =* 0.5 means more vaccination effort and more efficient local vaccination of susceptibles within the vaccine radius (i.e. “slow-and-steady”) compared to a fast expansion at rate *μ* = 50 (i.e. “fast-and-free”). We ran these simulations under the condition of “timely response”, for which both transmission and vaccine response begin at time step 0. The model is parameterized using the following values: time step *τ =* 0.1, transmission rate *δ =* 2, recovery rate *ρ* = 0.2, vaccine intensity *ε* = 20, and interaction scale *α =* 1 (see Supplementary Information for sensitivity of results to these parameters). We also performed an analogous set of simulations under the condition of a “delayed response”, where vaccine response begins 10 time steps (i.e. 10*τ* ∙ (*τ*/*ρ*)^−1^ = 2 epidemic generations) after the start of transmission. Assuming that distributing a vaccination response more quickly incurs a larger logistical cost, we define the optimum rate as the slowest rate *μ* that achieves the minimal number of infected individuals. Sensitivity analyses are performed by re-running the model after reducing and doubling the values of *δ*, *ε*, and *α* (S3–S6 Figs).

## Results

**Fig 2:**
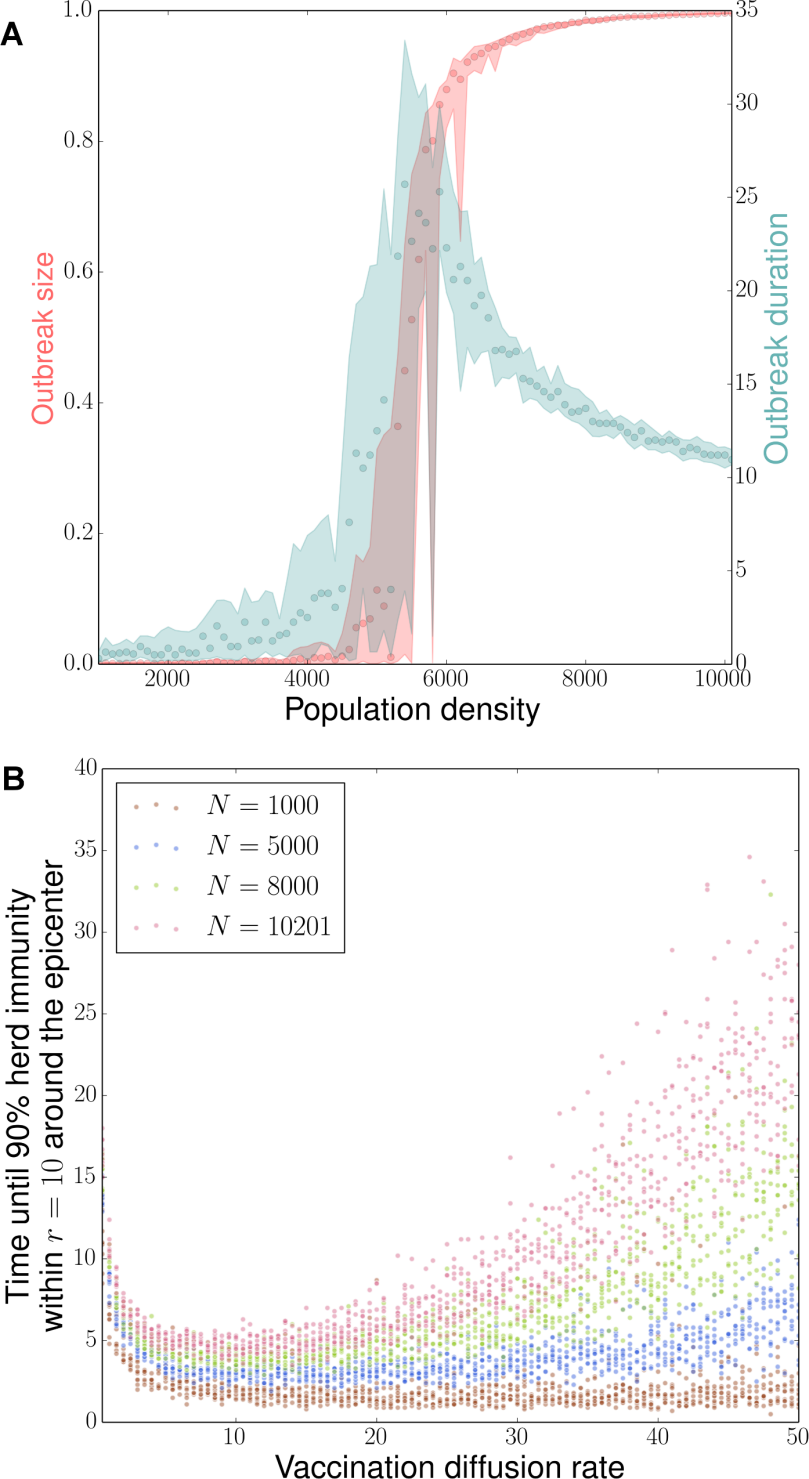
Density-dependence in transmission and vaccination processes. a) Fraction of population infected, i.e. outbreak size (red) and duration (blue) as a function of population density in the absence of response effort. 60 density-specific simulation replicates are run until transmission opportunity ceases, each replicate is initialized with a central infected individual and a randomized population of susceptibles. Time step *τ =* 0.1, transmission rate *δ =* 2, recovery rate *ρ* = 0.2, and interaction scale *α =* 1. Circles indicate the median values of both measures; the lower and upper bounds respectively represent the 20^th^ and 80^th^ percentile values. b) Time required for different vaccination strategies to reach 90% herd immunity within a circle of radius *r* = 10 around the control epicenter in the absence of disease. In a disease-free population of density *N*, 10 replicates are simulated for each value of the vaccination diffusion rate *μ*. Time step *τ =* 0.1 and vaccine intensity *ε* = 20.

In the absence of vaccination response, our model reflects density-dependence in the transmission process as expected (Fig 2A). At low density *N* < 5000, the outbreak is self-contained for this set of transmission parameters; as there are large distances between individuals, transmission occurs infrequently and the disease may quickly burn out based on recovery rate alone (indicated by the short outbreak durations and low outbreak sizes on the left side of Fig 2A). As density increases, neighbor interactions increase, and the infection is able to linger in population patches by means of episodic infection events until it becomes an epidemic after a period of slow and stuttering advancement; thus both outbreak size and duration increase at intermediate densities (Fig 2A). However, above a critical density threshold (*N* = 5500), infection events begin to occur at accelerated rates, fueling larger outbreaks in shorter amounts of time. When *N* > 7000, the infection is able to spread even more rapidly through a global cluster of individuals, allowing relatively fewer susceptibles to avoid infection. Therefore, we can infer that, if response measures are designed to control the spread of infection and the latter differs in efficiency across population densities, then proper response measures should also be density-dependent.

In the absence of disease transmission, the time required to achieve strong herd immunity ahead of the outbreak varies nonlinearly with vaccination diffusion rate in a pattern that reflects both spatial constraints and negative density-dependence in the response process (Fig 2B). When the population (here, susceptible) at a given location is low, the mean waiting time to vaccination per unit effort is short. For a given focal area, regardless of population density, increasing vaccination diffusion rate from the lowest level results in faster time to local herd immunity. For the lowest population densities (e.g. *N* = 1000), very few individuals must be immunized per unit area to achieve herd immunity and there is no cost to rapid diffusion; even effort spread as thin as possible is sufficient to deplete susceptibles quickly. As population density increases, e.g. *N* > 1000, a larger number must be vaccinated to achieve the local herd immunity threshold and thus the time needed to achieve herd immunity (that is, a given proportion of susceptibles immunized) in a focal area scales positively with population density *N*. As vaccination diffusion rate increases beyond an intermediate level, a larger fraction of the fixed effort is allocated outside the focal area, and the waiting time to vaccinate enough individuals to achieve herd immunity gets longer. Thus, there is a direct cost to any given focal area of rapid vaccination diffusion because of effort distributed elsewhere.

Comparison of Fig 2A and 2B indicates a tradeoff between transmission and control potentials when managing outbreaks in a moderate-to-highly dense population: disease spreads broadly and rapidly as a result of increased population density; however, focal populations near the outbreak epicenter are more difficult to protect in time, especially when the response process is driven by a high diffusion rate. In an alternative, less realistic scenario where the vaccination process is unaffected by density constraints, rapid diffusion to overtake the outbreak can be applied at no cost–herd immunity is possible in key areas despite a rapid outflow of local vaccination effort; therefore, the control objective is optimized with a maximization of the vaccination diffusion rate. By accounting for the negative density-dependence of the control process, our model introduces an exponential cost to higher diffusion in the form of a waiting time. Successful outbreak control, therefore, is implicitly contingent on a balance between vaccination coverage and local efficiency. The combination of the two opposing density effects suggests the existence of optimal vaccination strategies that are moderately diffusive and context-dependent.

**Fig 3:**
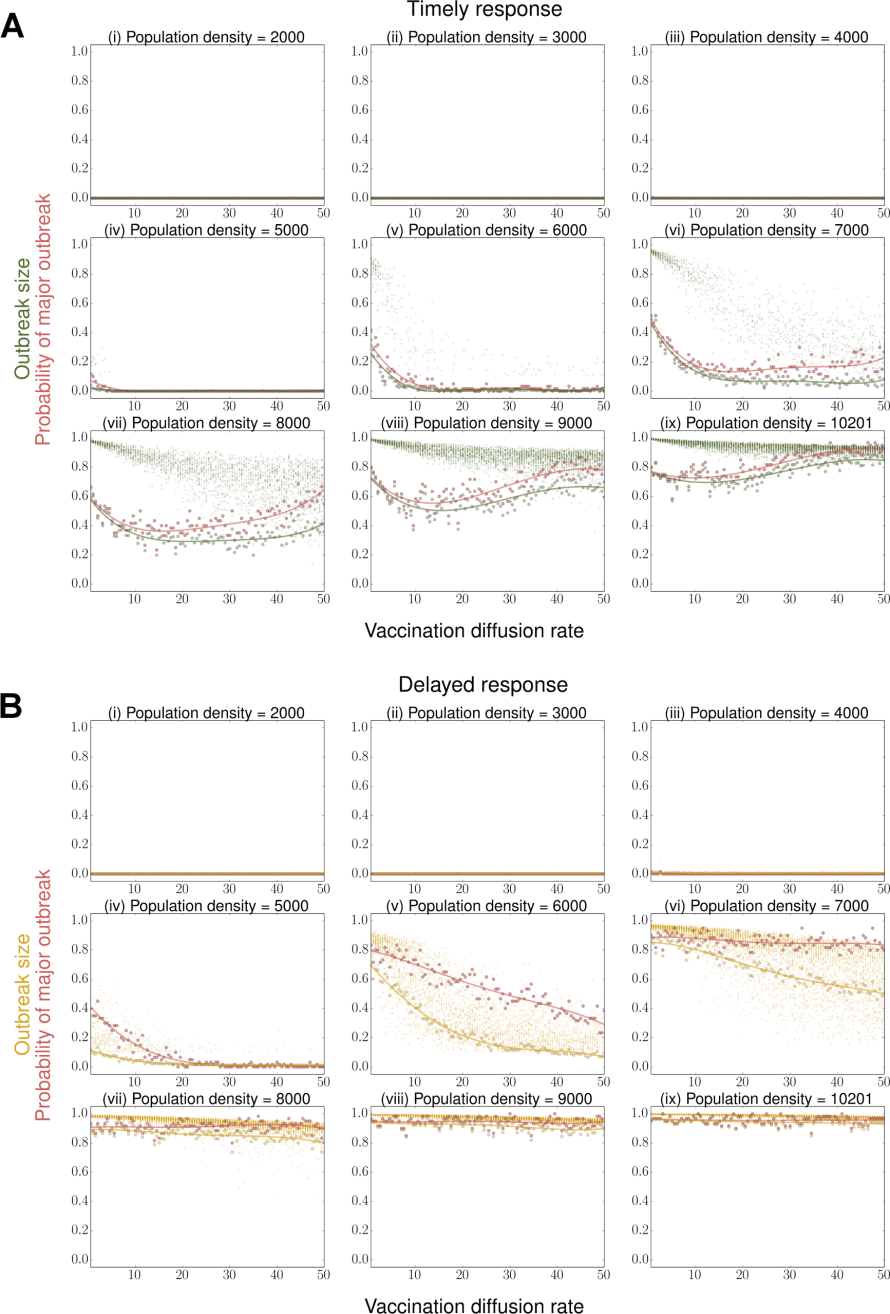
Proportions of the population infected at the end of an outbreak, conditional on the selection of vaccination strategy under different response times. Given population density, 60 simulation replicates were run for each vaccination diffusion rate *μ* under (A) a timely response, i.e. implemented at *t* = 0, and (B) a delayed response that lags behind the outbreak for 2 epidemic generations, i.e. implemented 10 time steps later. Time step *τ =* 0.1, transmission rate *δ =* 2, recovery rate *ρ* = 0.2, vaccine intensity *ε* = 20, and interaction scale *α =* 1. Large circles represent 1) the proportions of simulation replicates that result in a *major* outbreak event (red), defined by a threshold of ≥10% of total population infected, and 2) the expected proportional outbreak sizes of all simulation replicates under timely (green) and delayed (yellow) responses. Both (1) and (2) are fitted using 4^th^ order polynomial regression curves to smooth out simulation noise. Small circles of corresponding colors show the sizes of all major outbreaks in the events of occurrence.

We first consider a timely vaccination response, for which vaccination begins at the same time as the outbreak. At low population densities (*N* < 5000), infection is unlikely to spread (Fig 2A), consequently, a majority of the population will be uninfected regardless of which *μ* defined vaccination strategy is implemented (Fig 3A:i-iii); outbreak durations are short-lived after the initial case of infection (see S1 Fig).

**Fig 4:**
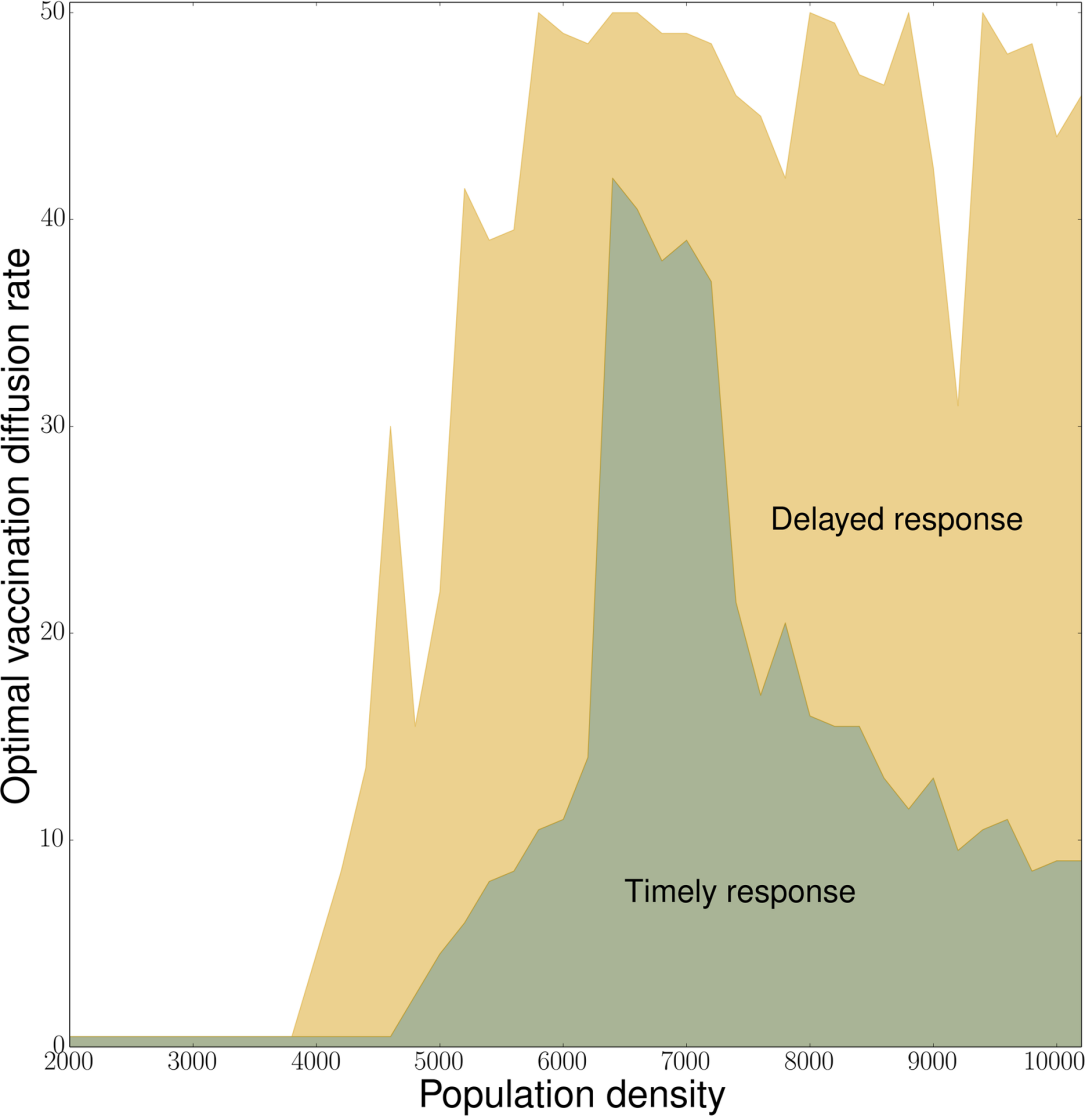
Optimal vaccination diffusion rates as a function of population density for a timely response (implemented at *t* = 0) and a delayed response of two epidemic generations (10 time steps later). Population densities are simulated from 2000 to 10201 at intervals of 200. Time step *τ =* 0.1, transmission rate *δ =* 2, recovery rate *ρ* = 0.2, vaccine intensity *ε* = 20, and interaction scale *α =* 1. The optimal diffusion rate indicates the minimum vaccination diffusion rate *μ* that minimizes the expected outbreak size for a given population density, computed from 60 simulation replicates for each value of *μ*.

Within the density range where infection readily establishes and spreads (Fig 2A), the effectiveness of a response strategy depends on its capacity to stay ahead of the outbreak. At intermediate population densities (5000 ≤ *N* ≤ 7000), our simulations show that, under low vaccination diffusion rates, herd immunity cannot be established quickly enough around new infection foci, thus allowing the remaining infections to spread into the surrounding area that has not yet achieved high proportion vaccinated. As a result, these strategies increase the probability of *major* outbreak events, defined as final infection prevalence ≥10% (we chose this threshold as it clearly divides the two modes of the outbreak size distribution between small outbreaks characterized by stuttering chains of infection and broad outbreaks, see S2 Fig), and widespread epidemics when they occur (Fig 3A:iv-vi). In comparison, with increased rates of vaccination diffusion, the response effort proceeds ahead of the outbreak and has time to establish strong herd immunity farther from the epicenter (Fig 2B). These “faster-and-freer” strategies are therefore more effective at reducing the probability of major outbreaks and their sizes (Fig 3A:iv-vi). Because the marginal reduction in expected outbreak size converges to zero as *μ* increases, vaccination at an intermediate diffusion rate is sufficient to achieve the maximal benefit (Fig 3A:iv-vi, Fig 4). We computed optimal diffusion rate as $min({\arg\;\min}_{\mu_{i}}\left\{j(\mu_{i})|\mu_{i}=0.5,1,\ldots ,50\right\})$, where *j*(∙) refers to the rounded values of a 4^th^ order polynomial regression of the proportion infected as a function of vaccination diffusion rate in order to smooth over simulation noise.

At high population density (*N* > 7000), widespread epidemics are difficult to prevent regardless of the vaccination strategy chosen (Fig 3A:vii-ix); major outbreaks can occur with 95% probability and, when they do, they infect almost the entire population. Within these dense populations, vaccination effort deployed by “slow-and-steady” strategies are prone to be immediately outstripped by the rapidly spreading outbreak. “Fast-and-free” strategies, on the other hand, push vaccination effort well beyond the outbreak perimeter and administer vaccines to many more individuals in areas not yet reached by the outbreak. If the diffusion rate is too high, then the per unit vaccination effort is low and infection can continue to spread through susceptible individuals who remain in the vaccination queue. In contrast, intermediate vaccination rates (10 < *μ* < 20) achieve a balance between (a) staying ahead of the outbreak and (b) allocating sufficient effort to the core epidemic area to establish local herd immunity within the target control area. The effectiveness of these strategies is shown by their ability to reduce the probability of major outbreak events to 40%, which in turn minimizes the expected outbreak sizes.

We verified that intermediate vaccination diffusion rates are optimal to control outbreaks in moderately-to-highly dense populations for a range of interaction scale, transmission rate, and vaccine intensity parameters (see S3–S6 Figs). We performed separate simulations across the same set of vaccination strategies assuming a timely response, varying only the value of a single parameter. The population density at which the intermediate optimum arises, and the relative benefit of this optimum compared to the slowest and fastest distribution strategies are shown to vary. For instance, the lowest density at which an intermediate diffusion rate is optimal negatively correlates with both the interaction scale and transmission rate (S3 and S4 Figs). When we isolate the sensitivity runs simulated in populations where outbreaks can occur (51 out of 81 parameter and population density combinations where the minimal outbreak size cannot be achieved equally by all vaccination strategies (see Fig 3A:iv-ix)), the optimal strategy generally emerged at an intermediate value (S6 Fig). In a few exceptions (3 out of 51 parameter combinations), the optimum is minimized at diffusion rate *μ* = 0.5; all 3 of these reflect settings at the highest population density with the highest transmission rate or interaction scale considered. In no case was the highest vaccination rate optimal. The remaining optima tend to occur in the lower half of the vaccine distribution rates that we considered, suggesting that there is frequently a cost to distributing vaccination faster, or more broadly, than necessary.

We also optimized vaccination strategies when the response is delayed, lagging behind the outbreak by 2 epidemic generations. When population density *N* > 4000, the simulation always results in fewer individuals being protected (i.e. more are infected) than with a timely response under identical strategies (Fig 3B:iv-ix). However, at all densities we considered, the optimal rate of vaccination diffusion is always higher than for a timely response (Fig 4). For some densities, a delayed response can achieve a level of protection equivalent to a sub-optimal timely response. For example, at density *N* = 6000 (Fig 3B:v), if vaccination response is delayed by 2 epidemic generations, 80% of the population is expected to be protected by a strategy of *μ* = 20; the same result can be achieved by a strategy of *μ* = 1 with a timely response.

## Discussion

Here, we present a linked epidemic and vaccination distribution model that evaluates the performance of spatially explicit vaccination distribution strategies. The model integrates an agent-based SIRV simulation of a stochastic outbreak with a continuous-time diffusion model that describes the process of vaccination diffusion. Vaccination coverage is spatially constrained and individual vaccination rate depends both on the local density of susceptibles (vaccine demand, or need) and the area over which vaccination resources are distributed. We found that, for a timely vaccination response, the optimal rate of vaccination diffusion depends on population density. Under our model setting, at low densities (*N* < 5000), outbreaks typically self-terminate, independent of the rate at which vaccine is distributed; at intermediate densities (5000 < *N* < 8000), outbreaks may be limited as long as the distribution of vaccination is fast enough to stay ahead of disease spread. Here, intermediate and high diffusion rates are equivalent at limiting outbreak size, but intermediate diffusion rates are sufficient; thus, if there are logistical costs to a faster vaccination program (e.g. more transportation effort necessary) then an intermediate strategy that achieves the same outbreak size may be favored. At high densities (*N* > 8000), outbreak size is minimized by vaccination strategies of intermediate diffusion rates that balance the need to stay ahead of the outbreak and to achieve high enough local coverage around the disease epicenter to slow spread. In this regime, major outbreaks, if they occur, affect a significant fraction (generally *>* 0.4) of the population (Fig 3A). While rapid strategies always reduce the mean size of these major outbreaks, on average, they are suboptimal because, by spreading vaccination resources too thin, the probability of a major outbreak occurring is higher. Indeed, for vaccination diffusion rates above an intermediate threshold, the probability of major outbreaks spreading through the population that has yet to receive vaccination may equal or exceed that of the slowest vaccination diffusion rate. However, when response time lags behind the outbreak by multiple epidemic generations, the optimal strategy is to vaccinate as broadly and swiftly as possible regardless of the number of susceptibles on the landscape.

Based on sensitivity analysis, the optimality of intermediate rates of vaccination in high-density contexts appears to be a general pattern that emerges from the dynamic interactions between transmission and response processes. For all parameter combinations considered, we found a range of susceptible densities over which the outbreak size is minimized by vaccination diffusion at an intermediate rate. Thus, we find that the emergence of an intermediate optimum diffusion rate as population density increases was consistent; however, the population density at which this intermediate optimum emerges and the relative benefit of the optimum depends on the epidemiological context.

By modeling vaccination as a dynamic process, our results address the inherent constraints and tradeoffs in deploying control over space and time. Conventionally, management though vaccination is described in terms of the herd immunity threshold: the proportion of the population that needs to be immune in order to prevent epidemic spread. For instance, this threshold is 1 − 1/*R*_0_ in the classic mean-field models [38] and, similarly, network models of human contact assess conditions for stopping epidemics in terms of the necessary reduction of nodes [39]. Both classes of models investigate an important management question: what is the threshold proportion of susceptibles necessary to prevent an outbreak? However, these statements are made in terms of the equilibrium criteria to prevent epidemic spread and largely ignore the operational question of *how* to reach those control targets or *how* outbreak responders should be distributed. Thus, these classic control objectives ignore the potential for transient phenomena that may arise from the process of achieving those objectives. Here, we show that the transient dynamics of epidemic spread and vaccination during outbreak response can lead to counter-intuitive control recommendations.

By formalizing the spatial limitation and temporal (i.e. transient) dynamics of vaccine delivery in our model, we are able to find an optimal strategy in terms of the action to implement (e.g. diffuse vaccination effort at a specific rate) rather than an outcome to achieve (e.g. total coverage). Furthermore, our representation of an explicit vaccination process reveals the intrinsic tradeoff in the consequence of any action (i.e. area of coverage versus treatment intensity), such that vaccinating a population as extensively as possible can be suboptimal in dense demographic settings. If we consider that population density may change over time, for example when suburban, sparsely-inhabited areas grow, these results would further suggest that the optimal response strategy is likely to differ between outbreaks that happen at different stages of urban development, changing over time from “slow-and-steady” to intermediate rate vaccination.

An emergent pattern of our model is the effect of initial response time on the optimal vaccination diffusion strategy. As expected, timely response surpasses delayed response in terms of the fraction of the population protected under all density conditions; the distribution of outbreak sizes for a timely response is consistently lower than that for a delayed response. Interestingly, however, the recommendation from this model is that the best strategy for a timely response is different from that of a delayed response. In particular, a timely response performs best when vaccination is distributed more slowly, with more effort initially concentrated in the local vicinity of the outbreak epicenter, thus areas (or individuals) far from the epicenter indirectly benefit from the stronger effort to locally contain the outbreak. This containment was not possible with a delayed response in our simulations; therefore, the best option is to distribute protection as quickly as possible to the whole population. To the extent that the slower vaccination diffusion rate recommended for a timely response is less expensive to implement, our results would suggest that the outcome could be improved by allocating that savings to enhanced surveillance and rapid outbreak detection, yielding a smaller outbreak overall.

There have been analogous studies of control of pests and invasive species that have highlighted the benefit of focusing effort on the invasion front [40–41], thus providing indirect protection to areas not yet invaded. Here, because we require that vaccination be distributed in all areas under control, rapid diffusion quickly pushes vaccination response to the epidemic front, but high diffusion rates provide too little protection at the epidemic front to prevent continued spread.

To achieve mechanistic clarity, we wrote our model in a general form and made a number of simplifying assumptions regarding the response process. First, we fixed the center of vaccination effort to the initial disease epicenter throughout the simulation. Second, the total amount of vaccination resource is subject to a conservation constraint, such that the value of its spatial integral equals one at all time steps; thus, we have presumed that any type of depleting resource (e.g. vaccine, syringe) is promptly replenished during each transmission cycle and never increases, e.g. due to an influx of additional resources. Third, since we defined vaccination strategies only by constant diffusion rates, the distribution of response effort is insensitive to local context-dependent factors. This suggests an implicit vaccination priority in the campaign, one that always favors individuals close to the epicenter rather than targeting those in more remote, higher-risk areas. Fourth, we implemented response in homogenously distributed populations with low degrees of clustering.

While these assumptions are simplistic relative to logistical complexity of vaccination response, they do present a low-dimensional representation of key operational constraints that are seen in real vaccination campaigns. House-to-house oral polio vaccination campaigns are among the best documented [42–44]. Under this strategy, teams of vaccinators go from house to house vaccinating eligible children, thus, there is a fixed time cost per household which requires either more time or more vaccination teams to achieve high coverage in areas with larger populations. In practice, detailed microplanning can be used to efficiently prioritize areas and the movement of vaccination teams [45], but simple strategic models, such as presented here, could be used to evaluate candidate strategies. Many vaccines cannot be delivered through a house-to-house strategy because of either cold-chain restrictions or the need for trained vaccinators for injectable delivery; e.g. measles vaccine. These vaccines are often delivered through fixed post locations in population centers [33]. Even for these strategies, which rely on residents to travel to the vaccination centers, the performance of the campaign depends critically on social mobilization, whereby canvassers go house-to-house, to markets, to religious centers, etc. to alert the population to the vaccination activity and the location of the vaccinators. While we do not explicitly account for vaccinators and canvassers, the same phenomenological pattern would be expected: more households take longer time or more canvassers to reach, thus resulting in the simple density-dependent relationship in the time until a target proportion (e.g. Fig 2B) of the population receives information about a campaign and travels to the fixed post.

In all scenarios presented here, we assume that the same vaccine delivery strategy is applied in all areas, which the model expresses using a constant diffusion coefficient for the deployment of vaccination effort. This describes a campaign that covers a fixed distance in random direction per unit time, which may be difficult to achieve in more remote regions. However, in practice, it is common to use mixed strategies, with fixed post delivery in dense urban centers and mobile strategies in more rural areas [33]. Further, many strategies are reactive to local needs, i.e. rapidly deploying vaccine to outbreak foci or to areas at high risk of epidemic spread (e.g. Coup de Poing implemented by MSF Belgium’s Pool d’Urgence Congo [46]). Similarly, areas that are easily accessible, or have high population density, may benefit from economies of scale and the fixed costs (in terms of money or time) may then be trivial compared to the large beneficiary population. The framework we have presented here could be extended to formally address these mixed, reactive strategies and their benefits in heterogeneously distributed populations, including prioritized response rather than simple radial diffusion of effort.

Future extensions of our model where the existing assumptions are relaxed can be used to explore vaccination delivery systems of increased realism and complexity. These developments are technically approachable using our current framework, which has an analogous form to classic models of animal home range and territory formation. Equations that combine diffusion and advection processes have been used to describe either attractive or avoidant behavioral responses of territorial mammals to local stimuli, such as resource availability [47] or scent-marks of neighborhood competitors [48]. New deployment strategy of healthcare personnel, like the space use patterns that result from animal movement, can be mathematically expressed in a similar way. The departure from constant vaccination diffusion rate allows us to explore the effects of locally reactive, “smart” vaccination strategies that, for instance, prioritize distribution of resource to high-risk (i.e. densely clustered) areas while forgoing areas of low protective benefit. Additionally, some animals are known to temporally shift their territorial centroids (“den sites”) along a particular habitat or topological gradient [49–50]; this dynamic feature could likewise apply to a vaccination strategy with a mobile control center that periodically relocates toward higher risk areas. In reality, local provisions of vaccination effort do not always adhere to the prescribe strategy, especially when there exists spatial heterogeneity in transportation structure, such as in the number of roadways in rural areas. These limitations to human movement can be expressed as a set of geographically dependent diffusion rates that reflect the level of local infrastructural development. While extensions of our model into reactive and complex control strategies can make the task of optimization more difficult due to the increased computational cost of evaluating over a larger, more stochastic set of outcomes, this drawback can be mitigated using novel system-level methods to reduce pieces of the agent-based simulations to mathematical expressions that allow standard optimization analysis [51]. In this paper, however, we have purposefully simplified many details of supply chain logistics in exchange for a simple, heuristic description of vaccination process. Problems of operational research in general are nevertheless central to real-world feasibility of outbreak response [52–53], thus should be considered as an integral component of epidemic problems at large. We intend to further pursue integrations of transmission and response processes using our model framework in order to better understand the complex, high-dimensional relationships between optimal vaccination strategies and both demographic and epidemiological dynamics.

Our study represents a simple framework for studying disease response under logistical constraints. We showed that vaccination strategies informed by an operational understanding of vaccine allocation are critical to optimizing public health objectives. Future work that builds on our implementation of a spatially constrained vaccination process is particularly relevant for reliably predicting the consequence of intervention campaigns, as it would identify necessary tradeoffs and prioritizations in vaccine distribution.

## Supporting information

S1 Appendix Overview, Design concepts and Details (ODD) protocol. (DOCX)

S1 Fig Timeframe spanned by an outbreak, conditional on the selection of vaccination strategy under different response times. Given population density, 60 simulation replicates were run for each vaccination diffusion rate *μ* under (A) a timely response, i.e. implemented at *t* = 0, and (B) a delayed response that lags behind the outbreak for 2 epidemic generations, i.e. implemented 10 time steps later. Time step *τ =* 0.1, transmission rate *δ =* 2, recovery rate *ρ* = 0.2, vaccine intensity *ε* = 20, and interaction scale *α =* 1. Squares represent the expected outbreak duration, i.e. length of timeframe, of all simulation replicates under timely (green) and delayed (yellow) responses. The means are fitted using 4^th^ order polynomial regression curves to smooth out simulation noise.
(TIF)

S2 Fig Histogram of outbreak sizes ≥ 0.005. Population densities are simulated from 2000 to 10201 at intervals of 200. Time step *τ =* 0.1, transmission rate *δ =* 2, recovery rate *ρ* = 0.2, vaccine intensity *ε* = 20, and interaction scale *α =* 1. For each population density, 60 simulation replicates were run for each vaccination diffusion rate 0.5 < *μ <* 50 at intervals of 0.5 under a timely response, i.e. implemented at *t* = 0. Majority (73.37%) of all simulations produced outbreak sizes < 0.005; the remaining results still clearly show a bimodal distribution, where the breakpoint exists at approximately 0.1 (dashed line). This threshold value determines our definition of a *major* outbreak event.
(TIF)

S3 Fig Sensitivity of density-dependent optimal vaccination rate for three levels of interaction scale, conditional on timely vaccination. (A, B) show, respectively, the fraction of the population infected at the end of an outbreak under a timely response times, i.e. implemented at *t* = 0, and optimal vaccination diffusion rates as a function of population density, both for interaction scale *α* = 0.75; (C, D) the same for interaction scale *α* = 1; (E, F) the same for interaction scale *α* = 2. Time step *τ =* 0.1, transmission rate *δ =* 2, recovery rate *ρ* = 0.2, and vaccine intensity *ε* = 20. In (A, C, E), given a population density, 60 simulation replicates were run for each vaccination diffusion rate *μ*. Large circles represent 1) the proportions of simulation replicates that result in a *major* outbreak event (red), defined by a threshold of ≥10% of total population infected, and 2) the expected outbreak sizes of all simulation replicates. Both (1) and (2) are fitted using 4^th^ order polynomial regression curves. Small circles show the sizes of all major outbreaks in the events of occurrence. In (B, D, F), the optimal diffusion rate indicates the minimum vaccination diffusion rate *μ* that minimizes the expected outbreak size for a given population density.
(TIF)

S4 Fig Sensitivity of density-dependent optimal vaccination rate for three levels of transmission rate, conditional on timely vaccination. (A, B) show, respectively, the fraction of the population infected at the end of an outbreak under a timely response times, i.e. implemented at *t* = 0, and optimal vaccination diffusion rates as a function of population density, both for transmission rate *δ* = 1; (C, D) the same for transmission rate *δ* = 2; (E, F) the same for transmission rate *δ* = 4. Time step *τ =* 0.1, recovery rate *ρ* = 0.2, vaccine intensity *ε* = 20, and interaction scale *α =* 1. In (A, C, E), given a population density, 60 simulation replicates were run for each vaccination diffusion rate *μ*. Large circles represent 1) the proportions of simulation replicates that result in a *major* outbreak event (red), defined by a threshold of ≥10% of total population infected, and 2) the expected outbreak sizes of all simulation replicates. Both (1) and (2) are fitted using 4^th^ order polynomial regression curves. Small circles show the sizes of all major outbreaks in the events of occurrence. In (B, D, F), the optimal diffusion rate indicates the minimum vaccination diffusion rate *μ* that minimizes the expected outbreak size for a given population density.
(TIF)

S5 Fig Sensitivity of density-dependent optimal vaccination rate for three levels of vaccine intensity, conditional on timely vaccination. (A, B) show, respectively, the fraction of the population infected at the end of an outbreak under a timely response times, i.e. implemented at *t* = 0, and optimal vaccination diffusion rates as a function of population density, both for vaccine intensity *ε* = 10; (C, D) the same for vaccine intensity *ε* = 20; (E, F) the same for vaccine intensity *ε* = 40. Time step *τ =* 0.1, transmission rate *δ =* 2, recovery rate *ρ* = 0.2, and interaction scale *α =* 1. In (A, C, E), given a population density, 60 simulation replicates were run for each vaccination diffusion rate *μ*. Large circles represent 1) the proportions of simulation replicates that result in a *major* outbreak event (red), defined by a threshold of ≥10% of total population infected, and 2) the expected outbreak sizes of all simulation replicates. Both (1) and (2) are fitted using 4^th^ order polynomial regression curves. Small circles show the sizes of all major outbreaks in the events of occurrence. In (B, D, F), the optimal diffusion rate indicates the minimum vaccination diffusion rate *μ* that minimizes the expected outbreak size for a given population density.
(TIF)

S6 Fig Stacked histogram of optimal vaccination diffusion rates under population densities capable of outbreaks. Results are collected and tallied from three sets of sensitivity analysis (S3–S5 Figs), excluding those under density conditions whereby an infection cannot establish and spread (indicated by optimal diffusion rate *μ =* 0.5 in low-density context). Bars indicate the number of parameter combinations for which a specific diffusion rate was optimal. Colors indicate results from sensitivity runs with varying interaction scale (blue), transmission rate (green), and vaccine intensity (red), respectively.
(TIF)

S1 Video Movie of the timely vaccination response to an outbreak that is shown in Fig 1. Population is initialized with *N* = 10201 susceptibles, vaccination diffusion rate *μ* = 25, time step *τ =* 0.1, transmission rate *δ =* 2, recovery rate *ρ* = 0.2, vaccine intensity *ε* = 20, and interaction scale *α =* 1.
(MP4)
