# Supplementary material for: Logistical constraints lead to an intermediate optimum in outbreak response vaccination
Source: PLoS Comput Biol. 2018 May 23;14(5):e1006161. doi: 10.1371/journal.pcbi.1006161 (PMC5988332; doi:10.1371/journal.pcbi.1006161)
Supplement: S1 Appendix — (DOCX) [file pcbi.1006161.s001.docx]

**Overview, Design concepts and Details (ODD) protocol**

for

*Logistical constraints lead to an intermediate optimum in outbreak response vaccination*

by

Tao, Shea and Ferrari

1. *Purpose*

The purpose of the model is to understand how diffusing vaccination effort at different rates of diffusion from the outbreak epicenter affects the dynamics of disease spread and to evaluate the relative performance of different vaccination diffusion rates within populations of particular densities. Furthermore, the model aims to expand from conventional formulations of vaccination response in an SIRV model to a more realistic representation that includes logistical constraints that vary in space and time.

1. *Entities, state variables and scales*

The model entities include individuals and the environment of outbreak response, defined by the temporal and spatial distribution of vaccination effort. Individuals are immobile and are characterized by two state variables: discretized spatial location and disease status, which can be susceptible, infected, recovered, or vaccinated. Multiple individuals are allowed to reside at the same location. Once a susceptible is vaccinated or recovered, it remains so for the rest of the simulation, i.e. we assume no loss of acquired immunity.

The environment of vaccination response is characterized by a circular, Gaussian distribution of vaccination effort that diffuses from the outbreak epicenter, which is located at the center of the modeled region. The diffusive process is computed using partial differential equation solver, FiPy; the volume under the surface of vaccination effort is constrained to one inside the region’s outer boundaries with the application of Neumann boundary condition. The dynamical distribution of vaccination effort is controlled by a single state variable of constant diffusion rate.

The modeled region comprises 101 x 101 grid cells of length 1. The simulation is run over discrete time step of length 0.1.

1. *Process overview and scheduling*

Within each time step, an individual’s disease status can be updated by three possible modules processed in the following order: recovery, transmission, and then, if the duration of response delay has lapsed, vaccination. In the latter scenario, the distribution of vaccination effort diffuses radially and its surface values at all locations are numerically solved prior to recovery. Under each module, new disease statuses are stored until it has been executed upon all individuals, and then all are updated synchronously before the next module. The entire simulation ends once the number of infected individuals reaches zero. The code updates plots after each time step and captures the developing spatial patterns of both the outbreak and its response.

1. *Design concepts*

- *Basic Principles*

The model reproduces basic compartmental models of disease transmission and control (Anderson and May, 1992), allowing individuals to partially transition between four distinct disease statuses: susceptible, infected, recovered, and vaccinated. Unlike classic models built within this framework, here, the process of vaccination occurs neither at a constant rate nor homogenously over space. The concept of density dependence is applied at the level of transmission and vaccination submodels; it explicitly describes the connections between local population structure and the mean wait time after which a member individual becomes infected or vaccinated.

- *Emergence*

The shape of the vaccination performance curve under specific population density and response delay emerges from the interacting dynamics between transmission and response processes. All individuals regardless of disease status are passive such that they are incapable of using learning, sensing, or prediction to actively advance any measurable objective. As a result, individual-level adaptive and fitness-seeking behavior is excluded from the model. The diffusive distribution of vaccination effort also follows an *a priori* rate after the response delay and does not adapt to epidemiological conditions.

- *Interaction*

Two types of individual interactions are modelled implicitly: transmission rate increases with local density of infected, and vaccination rate decreases with local density of susceptibles.

- *Stochasticity*

All epidemiological parameters except recovery rate contribute to exponential probability functions of different event occurrences per time step. Recovery rate is interpreted as the probability of becoming no longer infected during a time step. The locations of initial susceptibles are randomized, because the focus of the model is to identify vaccination diffusion rates that are optimal for controlling populations characterized by their densities irrespective of their spatial distributions.

- *Observation*

For model testing, the spatial patterns of the outbreak (i.e. locations and disease statuses of all individuals) and its response (i.e. distribution of vaccination effort) were observed at each time step. For model analysis, three population-level variables were recorded at the end of the simulation: the population proportions of vaccinated and non-infected individuals, and the simulation duration.

*Initialization*

Each simulation was initialized with a single infected individual at the epicenter (50.5, 50.5), surrounded by *N*-1 randomly located susceptibles, where *N* defines the specified population density. The bivariate Gaussian distribution of vaccination effort has mean values at the same epicenter with variances of one.

*Input Data*

This model does not use input data to represent time-varying processes.

*Submodels*

*Recovery*: The probability of an individual recovery from infection during a time step is $\rho=0.2$, independent of demographic and epidemiological conditions.

*Transmission*: The risk for susceptible individuals to become infected per time step increases with the local force of infection, $I(\mathbf{x})$, defined as the convolution of current distribution of infected individuals and interaction kernel $K$, computed using Fast Fourier Transform (FFT). Interaction kernel $K$ has the form of a bivariate Gaussian distribution with mean zero and variance-covariance matrix $\alpha^{2}\mathbf{I}$, where $\alpha$ is interpreted as interaction scale and has default value of 1 (see parametric variation below, *Vaccination*). During each time step, a susceptible individual at $\mathbf{x}$ changes its disease state to infected with probability

$\varphi\left( \mathbf{x} \right)=1-exp[-\delta I\left( \mathbf{x} \right)]$,

where $\delta=2$ is defined as disease transmission rate. Simulations are also performed using $\delta$ values of 1 and 4 as part of the sensitivity analysis. New disease statuses for all susceptibles are updated synchronously at the end of the module. A new distribution of local force of infection is then computed for use in the next time step.

*Vaccination*: The probability for susceptible individuals to become vaccinated per time step is zero before simulation time $t$ exceeds specified duration of response delay $T'$. Afterwards, it increases with the local amount of vaccination effort, $H(\mathbf{x})$, but decreases with the local density of susceptibles, $S(\mathbf{x})$.$H(\mathbf{x})$ refers to the current distribution of vaccination effort, which, starting at $t>T'$, diffuses from the epicenter (50.5, 50.5) at constant rate $\mu$. $S(\mathbf{x})$ results from convolving using FFT the distribution of susceptible individuals and the same interaction kernel $K$ used in the transmission module with interaction scale $\alpha=1$. The effects of shorter and longer interactions scales with values of 0.75 and 2 are explored for the purpose of sensitivity analysis. During each time step, a susceptible individual at $\mathbf{x}$ changes its disease state to vaccinated with probability

$$\omega\left( \mathbf{x} \right)=1-\exp\left[ -\varepsilon\frac{H\left( \mathbf{x} \right)}{S\left( \mathbf{x} \right)} \right],$$

where $\varepsilon=20$ is a scaling parameter interpreted as the vaccination intensity. The value of $\varepsilon$ is halved and doubled in additional simulations for sensitivity analysis. New disease statuses for all susceptibles are updated synchronously at the end of the module. A new distribution of local susceptible density is computed for use in the next time step. Note that scheduled update of $S(\mathbf{x})$ at the end of vaccination module assumes that removals of susceptibles from the previous transmission module does not shorten mean wait time for remaining susceptibles to be vaccinated during the same time step. In other words, an individual may concurrently develop infection and still contribute to its local demand for vaccination effort for a limited period.

Table 1 – Overview of model processes and parameters

| Parameter | Value |
| --- | --- |
| Population density | 2,000-10,201 |
| Interaction scale | 1 |
| Recovery |  |
| Recovery rate | 0.2 |
| Transmission |  |
| Transmission rate | 2 |
| Vaccination |  |
| Vaccination intensity | 20 |
| Vaccination diffusion rate | 0.5-50 |
| Time lapsed before diffusion of vaccination effort |  |
| Timely response | 0 |
| Delayed response | 10 |

References

Anderson R, May R. Infectious diseases of humans: dynamics and control. Oxford: Oxford University Press. 1992.
