# Supplementary figures and images for: Logistical constraints lead to an intermediate optimum in outbreak response vaccination
Source: PLoS Comput Biol. 2018 May 23;14(5):e1006161. doi: 10.1371/journal.pcbi.1006161 (PMC5988332; doi:10.1371/journal.pcbi.1006161)

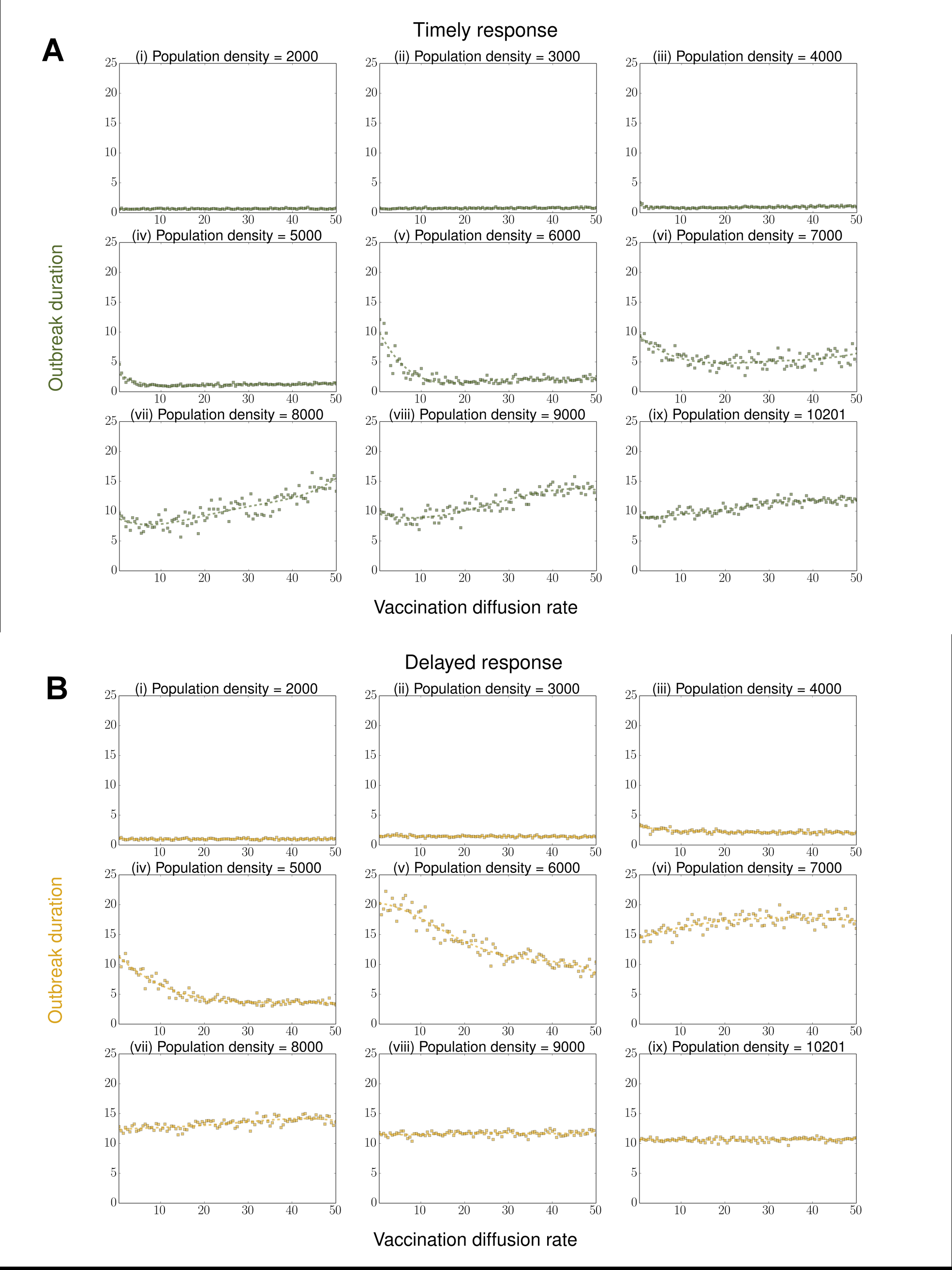

Supplement: S1 Fig — Given population density, 60 simulation replicates were run for each vaccination diffusion rate μ under (A) a timely response, i.e. implemented at t = 0, and (B) a delayed response that lags behind the outbreak for 2 epidemic generations, i.e. implemented 10 time steps later. Time step τ = 0.1, transmission rate δ = 2, recovery rate ρ = 0.2, vaccine intensity ε = 20, and interaction scale α = 1. Squares represent the expected outbreak duration, i.e. length of timeframe, of all simulation replicates under timely (green) and delayed (yellow) responses. The means are fitted using 4th order polynomial regression curves to smooth out simulation noise. (TIF) [file pcbi.1006161.s002.tif]

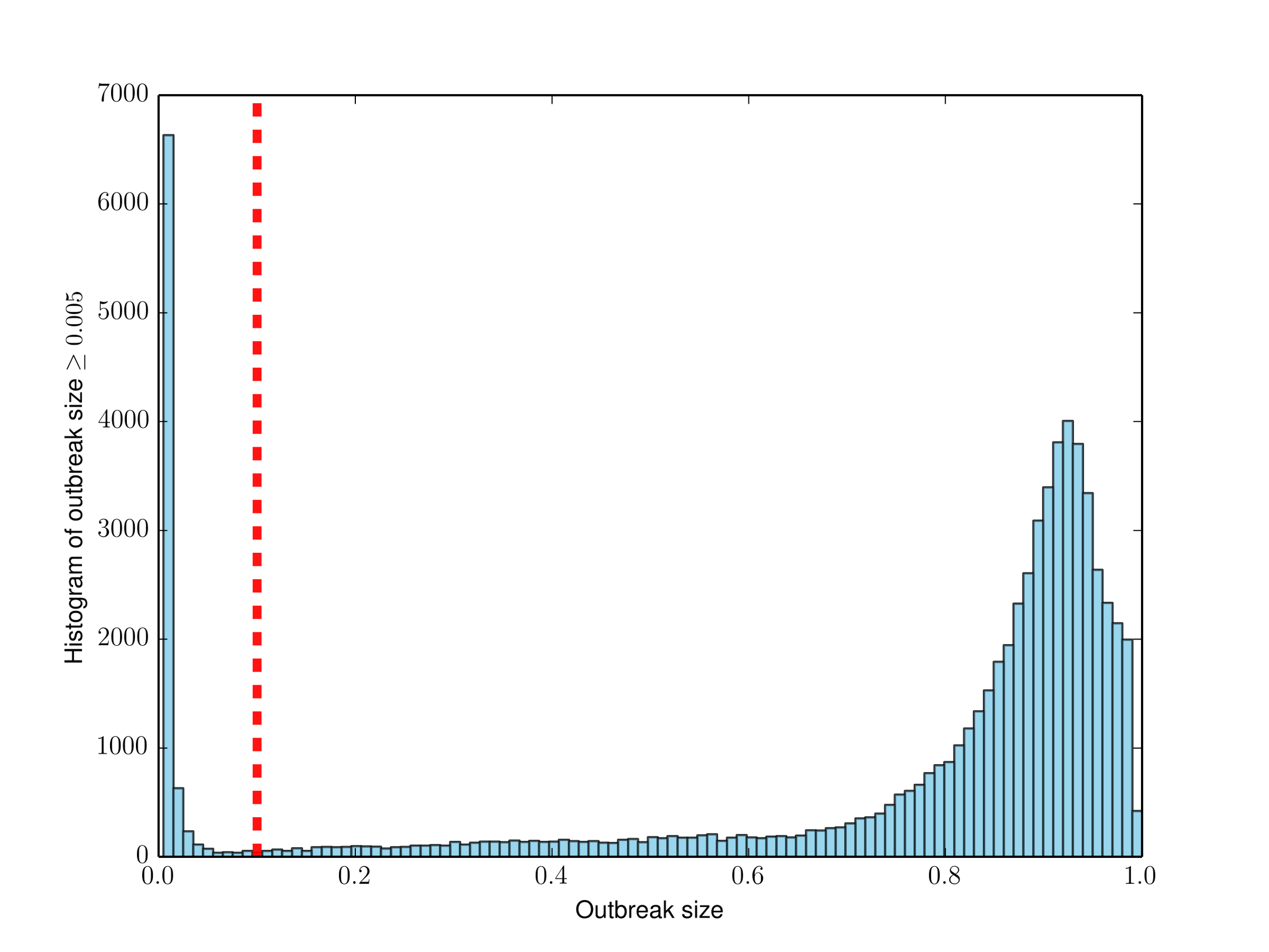

Supplement: S2 Fig — Population densities are simulated from 2000 to 10201 at intervals of 200. Time step τ = 0.1, transmission rate δ = 2, recovery rate ρ = 0.2, vaccine intensity ε = 20, and interaction scale α = 1. For each population density, 60 simulation replicates were run for each vaccination diffusion rate 0.5 < μ < 50 at intervals of 0.5 under a timely response, i.e. implemented at t = 0. Majority (73.37%) of all simulations produced outbreak sizes < 0.005; the remaining results still clearly show a bimodal distribution, where the breakpoint exists at approximately 0.1 (dashed line). This threshold value determines our definition of a major outbreak event. (TIF) [file pcbi.1006161.s003.tif]

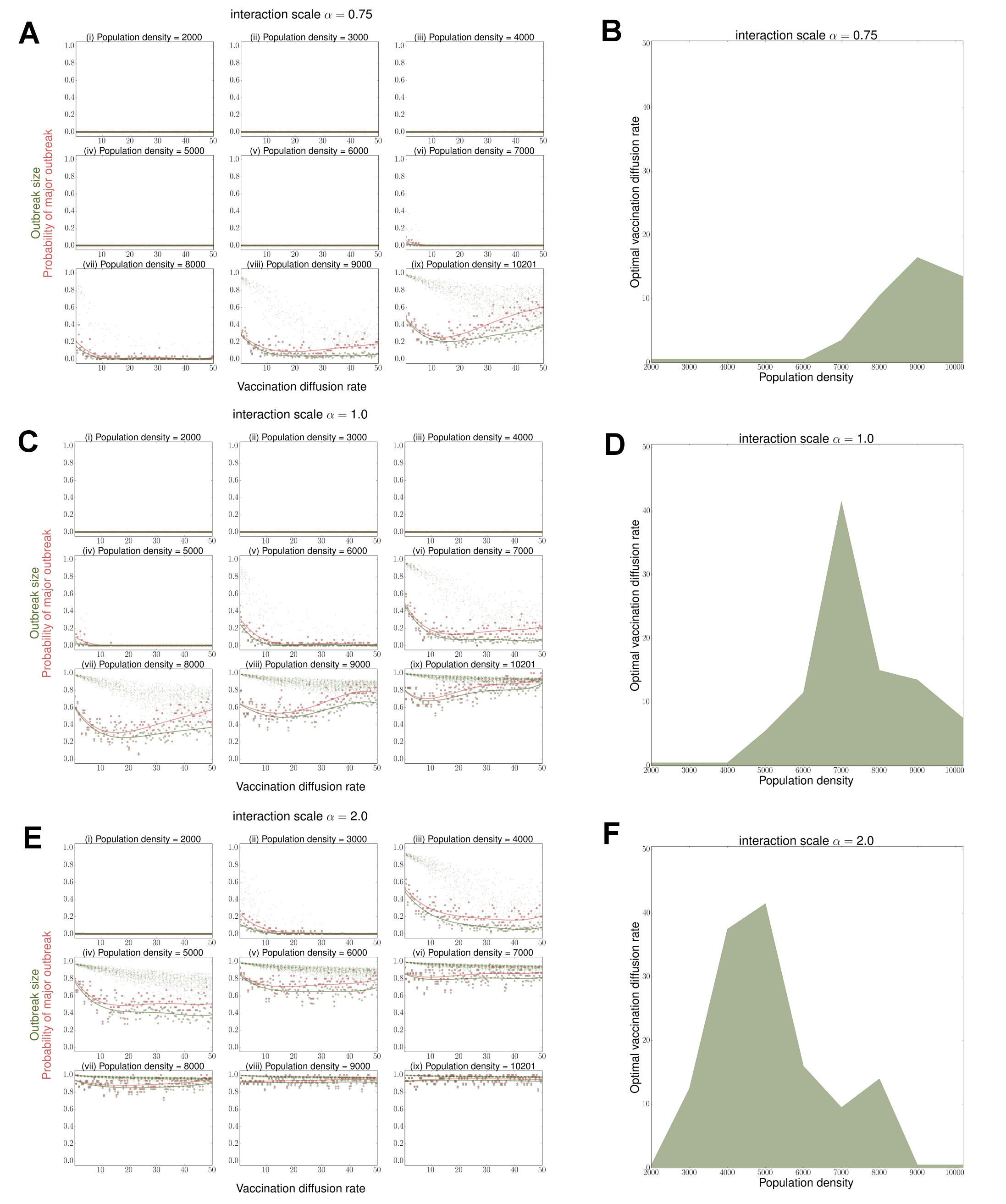

Supplement: S3 Fig — (A, B) show, respectively, the fraction of the population infected at the end of an outbreak under a timely response times, i.e. implemented at t = 0, and optimal vaccination diffusion rates as a function of population density, both for interaction scale α = 0.75; (C, D) the same for interaction scale α = 1; (E, F) the same for interaction scale α = 2. Time step τ = 0.1, transmission rate δ = 2, recovery rate ρ = 0.2, and vaccine intensity ε = 20. In (A, C, E), given a population density, 60 simulation replicates were run for each vaccination diffusion rate μ. Large circles represent 1) the proportions of simulation replicates that result in a major outbreak event (red), defined by a threshold of ≥10% of total population infected, and 2) the expected outbreak sizes of all simulation replicates. Both (1) and (2) are fitted using 4th order polynomial regression curves. Small circles show the sizes of all major outbreaks in the events of occurrence. In (B, D, F), the optimal diffusion rate indicates the minimum vaccination diffusion rate μ that minimizes the expected outbreak size for a given population density. (TIF) [file pcbi.1006161.s004.tif]

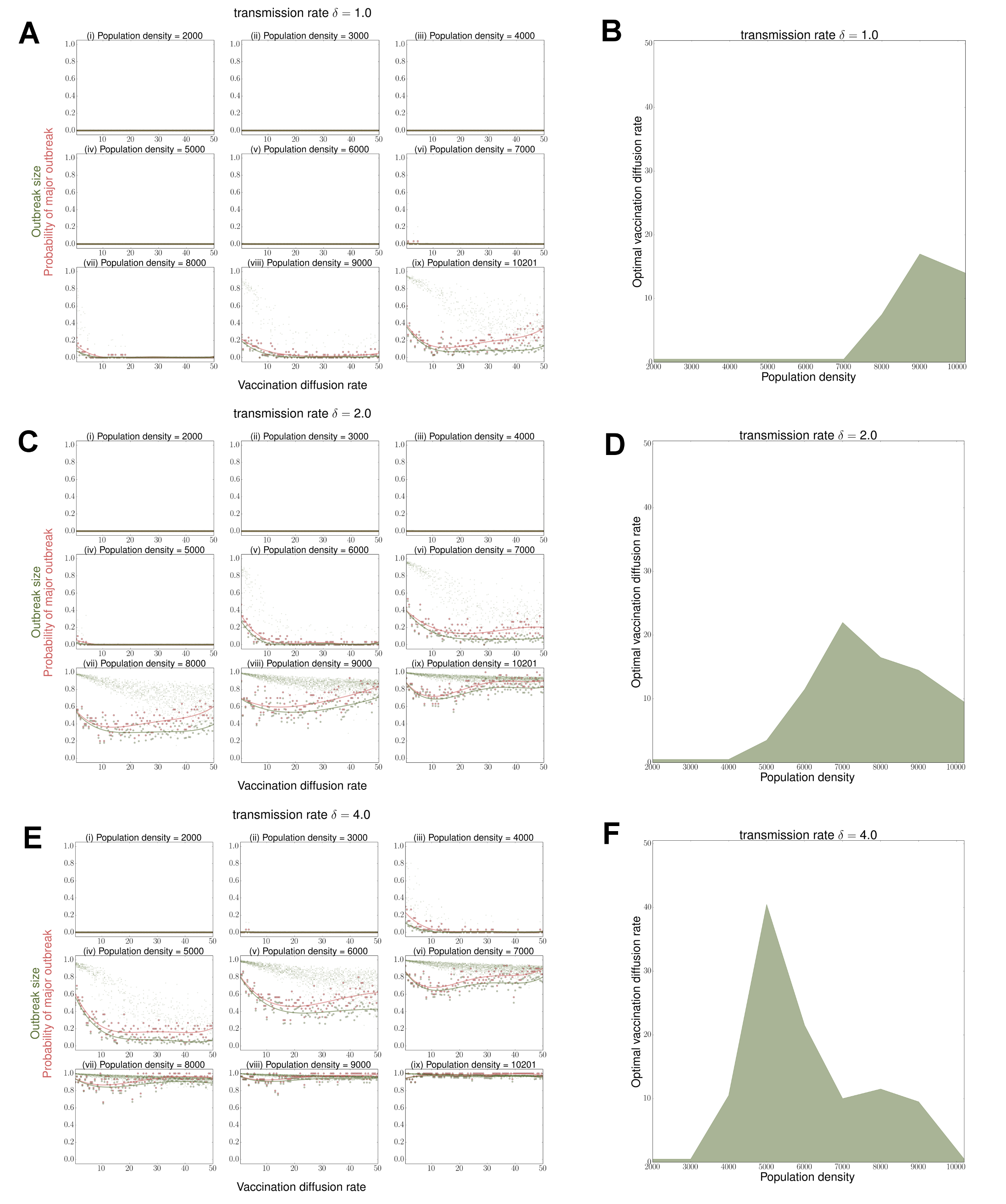

Supplement: S4 Fig — (A, B) show, respectively, the fraction of the population infected at the end of an outbreak under a timely response times, i.e. implemented at t = 0, and optimal vaccination diffusion rates as a function of population density, both for transmission rate δ = 1; (C, D) the same for transmission rate δ = 2; (E, F) the same for transmission rate δ = 4. Time step τ = 0.1, recovery rate ρ = 0.2, vaccine intensity ε = 20, and interaction scale α = 1. In (A, C, E), given a population density, 60 simulation replicates were run for each vaccination diffusion rate μ. Large circles represent 1) the proportions of simulation replicates that result in a major outbreak event (red), defined by a threshold of ≥10% of total population infected, and 2) the expected outbreak sizes of all simulation replicates. Both (1) and (2) are fitted using 4th order polynomial regression curves. Small circles show the sizes of all major outbreaks in the events of occurrence. In (B, D, F), the optimal diffusion rate indicates the minimum vaccination diffusion rate μ that minimizes the expected outbreak size for a given population density. (TIF) [file pcbi.1006161.s005.tif]

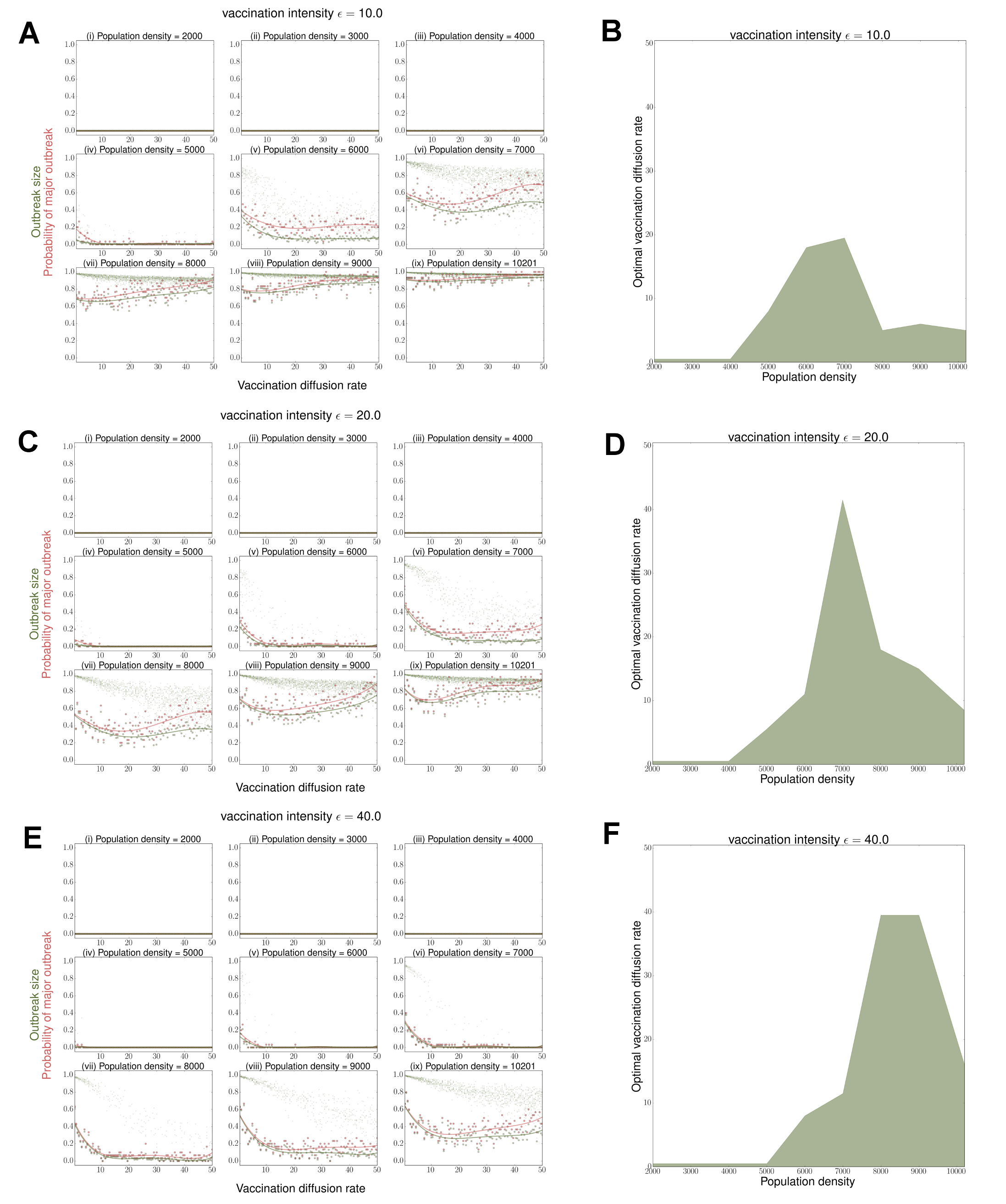

Supplement: S5 Fig — (A, B) show, respectively, the fraction of the population infected at the end of an outbreak under a timely response times, i.e. implemented at t = 0, and optimal vaccination diffusion rates as a function of population density, both for vaccine intensity ε = 10; (C, D) the same for vaccine intensity ε = 20; (E, F) the same for vaccine intensity ε = 40. Time step τ = 0.1, transmission rate δ = 2, recovery rate ρ = 0.2, and interaction scale α = 1. In (A, C, E), given a population density, 60 simulation replicates were run for each vaccination diffusion rate μ. Large circles represent 1) the proportions of simulation replicates that result in a major outbreak event (red), defined by a threshold of ≥10% of total population infected, and 2) the expected outbreak sizes of all simulation replicates. Both (1) and (2) are fitted using 4th order polynomial regression curves. Small circles show the sizes of all major outbreaks in the events of occurrence. In (B, D, F), the optimal diffusion rate indicates the minimum vaccination diffusion rate μ that minimizes the expected outbreak size for a given population density. (TIF) [file pcbi.1006161.s006.tif]

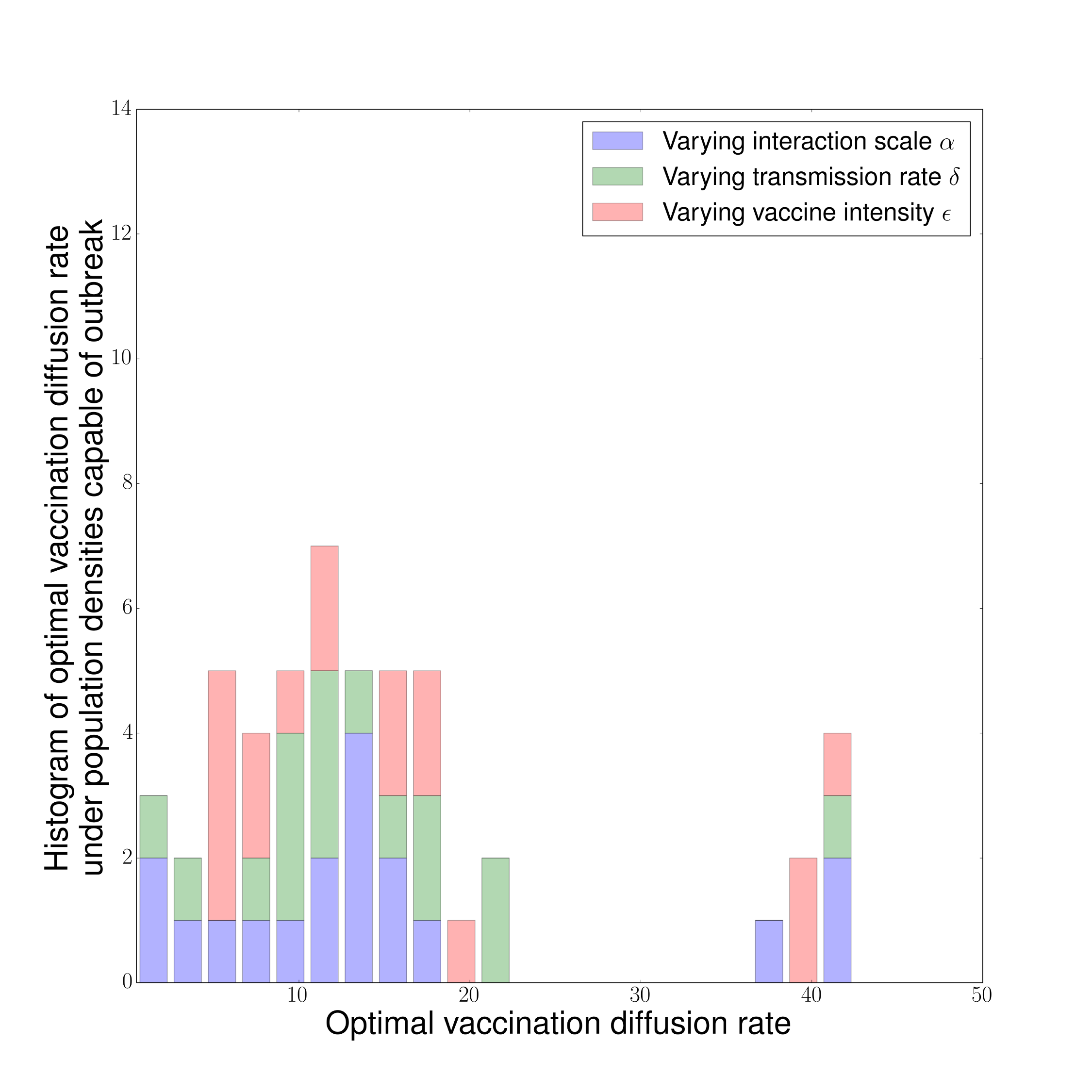

Supplement: S6 Fig — Results are collected and tallied from three sets of sensitivity analysis (S3–S5 Figs), excluding those under density conditions whereby an infection cannot establish and spread (indicated by optimal diffusion rate μ = 0.5 in low-density context). Bars indicate the number of parameter combinations for which a specific diffusion rate was optimal. Colors indicate results from sensitivity runs with varying interaction scale (blue), transmission rate (green), and vaccine intensity (red), respectively. (TIF) [file pcbi.1006161.s007.tif]
